# Supplementary material for: Optimizing cognitive and behavioral approaches for perinatal depression: A systematic review and meta-regression analysis
Source: Glob Ment Health (Camb). 2023 Mar 15;10:e22. doi: 10.1017/gmh.2023.8 (PMC10579678; doi:10.1017/gmh.2023.8)
Supplement: Supplementary file 1 [file S2054425123000080sup001.docx]

**Supplementary Table 1: Search strategy Condition/Population**

| **Concept** | **Search terms** |
| --- | --- |
| **Perinatal period** | (Perinatal[ti/ab] OR peripartum[ti/ab] OR antenatal[ti/ab] OR prenatal[ti/ab] OR pregnan*[ti/ab] OR postnatal[ti/ab] OR postpartum[ti/ab] OR postpartum[MeSH]) |
| **Depressive symptoms** | (depress*[ti/ab] OR “mood disorder*”[ti/ab] OR dysthym*[ti/ab] OR cyclothym*[ti/ab] OR depression[MeSH]) |
| **Type of Study** | (effectiveness[ti/ab] OR trial*[ti/ab] OR "clinical trial"[ti/ab] OR RCT[ti/ab] OR "randomized clinical"[ti/ab] OR implementation OR evaluation[ti/ab] OR "implementation science" OR feasibility[ti/ab] OR "program development"[ti/ab] OR Fidelity[ti/ab] OR appropriateness[ti/ab] OR acceptability[ti/ab] OR adoption[ti/ab] OR sustainability[ti/ab] OR penetration[ti/ab] OR appropriateness[ti/ab] OR cost-effectiveness) |
| **Interventions** | (“cognitive behavio* therapy”[ti/ab] OR “cognitive therapy”[ti/ab] OR “behavio* therapy”[ti/ab] OR problem-solving[ti/ab] OR “behave* activation” [ti/ab] OR “mindfulness based cognitive therapy” [ti/ab] OR “acceptance and commitment therapy”[ti/ab] OR “cognitive behavioral therapy”[MeSH]) |

**Supplementary Table 2: Types of cognitive behavioural therapies considered for inclusion in review** (Beck et al, 1979; Cuijpers et al, 2018; Kahl et al, 2012; Mazzucchelli et al, 2009)

| **Therapy** | **Definition** |
| --- | --- |
| **Cognitive Behaviour**  **Therapy (CBT)** | In CBT, the therapist targets the negative cognitive schema, leveraging the interconnection of thoughts and feelings with physical sensations and actions. The therapist tries to evaluate and identify patients’ negative and dysfunctional beliefs, then challenges and modifies them, thus trying to break the cycle between negative thoughts, behaviours, and actions. This approach is called cognitive restructuring. Unlike psychoanalytical approaches, the focus is on current problems rather than those in the past (Beck et al, 1979). By assigning homework and out-of-session activities, the emphasis is on building the capacity of the person to apply CB approaches in daily life. |
| **Behavioural activation**  **therapy (BAT)** | BAT interventions leverage the association between euthymic mood and participation in pleasant activities (Mazzucchelli et al, 2009). These interventions employ several strategies to achieve this, for example, utilizing ways for mood monitoring and increasing engagement in pleasurable or value-driven activities. The ultimate goal (Kahl et al, 2012) for BAT is to expose the patient to positive enforcement sources and achieve antidepressant action. |
| **Problem-solving**  **therapy (PST)** | We defined PST as a psychological intervention to build capacity for problem-solving among women with perinatal depression by identifying personal problems and promoting the use of problem-solving attitudes and skills (Cuijpers et al, 2018) |
| **Third wave cognitive**  **Behavioural therapies** | These are a heterogeneous and evolving group of psychotherapeutic methods, including but not limited to acceptance and commitment therapy, behavioral activation, cognitive behavioural analysis system of psychotherapy, dialectical behavioural therapy, metacognitive therapy, mindfulness-based cognitive therapy, and schema Therapy (Kahl et al, 2012). These therapies introduce new techniques to the prevalent “second wave” cognitive therapies. In contrast to classical cognitive therapies, the third wave therapies cautiously employ content-oriented (such as challenging dysfunctional thoughts), and promote the use of “first wave” behavioural principles such as operant conditioning (Kahl et al, 2012). |

**Supplementary Table 3: Taxonomy of elements and active ingredients utilized in the present review (Atif Rahman et al, 2018; Singla et al, 2017)**

| Elements | Active ingredients |
| --- | --- |
| **Non-specific elements** | - Involvement of family - Involvement of significant other - Active listening - Empathy - Collaboration - Inciting social support - Case management - Normalization - Eliciting commitment - Discussing advantages of treatment - Discussing barriers to treatment |
| **Techniques implemented by the therapist/delivery agent** | - Motivational enhancement - Praise - Roleplay - Behavioural contracting - Assigning homework - Interpersonal focus - Behavioural experiments - Motivational interviewing - Direct suggestions - Reviewing homework - Goal setting - Giving sick role - Empathy |
| **Interpersonal skills** | - Identifying affect - Identifying and eliciting social support - Communication skills - Assertiveness training - Assessing relationships |
| **Behavioural coping** | - Problem-solving - Relaxation - Exposure - Emotional regulation - Stress management - Decision making - Self-monitoring |
| **Reinforcement** | - Awarding positive behavior - Delay awards |
| **Parenting skills** | - Caregiver coping (e.g., management skills for the parents of children) - Parent-child Interaction Coaching (e.g., positive 1-on-1, attending to children modeling, etc) |
| **Psychoeducation** | - Birth procedures, adolescence or specific health areas of children e.g., nutrition, breastfeeding etc.) - nutrition - Breastfeeding - Sexual behaviour |
| **Cognitive coping** | - Identifying thoughts, behaviours, and their links (e.g., identifying negative thoughts, thought diary) - Cognitive restructuring (e.g., reattribution, weighing evidence, logical questioning) - Distraction - Self-talk - Self-praise - Mood monitoring - Mindfulness - Self-awareness |
| **Exercise** | - Aerobics - Non-aerobic exercise |
| **Nutrition** | - Macronutrients - Micronutrient - Eating behaviours |
| **Substance use** | - Alcohol consumption - Substance misuse |

**Supplementary Table 4: Definitions of therapeutic elements originally published as Rahman et al. 2018**

| **Sr #** | **Name of Skill** | **Definition** |
| --- | --- | --- |
| 1. | Involvement of family | The family members are involved in the intervention. |
| 2. | Involvement of significant other | The significant other or spouse is involved in the intervention. |
| 3. | Active listening | The skillful listens to the speaker with full concentration to understand what is being said [[1](#_bookmark0)]. |
| 4. | Collaboration | Working with others |
| 5. | Inciting social support | Providing insight of how others can provide help or support [[2](#_bookmark1)]. |
| 6. | Case management | Planning, facilitating and coordination of different options regarding the patient [[3](#_bookmark2)]. |
| 7. | Normalization | To communicate that the person’s experiences also happen to other people [[4](#_bookmark3)]. |
| 8. | eliciting commitment | Motivating the client to take active part in intervention. |
| 9. | Discussing advantages | Identifying advantages to take the intervention. |
| 10. | Discussing barriers | Identifying difficulties to take the intervention. |
| 11. | Identifying affect | Identifying feeling or emotion [[5](#_bookmark4)]. |
| 12. | identifying and eliciting social support | Providing consideration of how others could change their behaviour to offer the person help or support [[6](#_bookmark5)]. |
| 13. | Communication skills | These are set of skills which improve the dissemination, reception and exchange of information, opinions or ideas making sure that the intended message is completely understood by those involved [[7](#_bookmark6)]. |
| 14. | Assertiveness training | To train people for effective communication without being passive or aggressive. |
| 15. | Assessing relationships | To assess the relationships with people around. |
| 16. | Problem solving | To solve a problem by carefully defining problem and weighing different options to solve the problem. |
| 17. | Relaxation | To apply various techniques for relaxation. |
| 18. | Awarding positive behaviour | Applying different methods to encourage recurrence of a positive behaviour [[8](#_bookmark7)]. |
| 19. | Exposure | Confronting previously avoided objects, situations, unwanted thoughts or feelings while not avoiding or escaping from it [[9](#_bookmark8)]. |
| 20. | Emotional regulation | Tendency to manage and responding to emotional experience [[10](#_bookmark9)]. |
| 21. | Stress management | May involve a variety of techniques that do not target a specific behaviour but seek to reduce anxiety and stress [[6](#_bookmark5)]. |
| 22. | Decision making | Process of making choices by identifying decision, gathering information, and assessing alternative resolutions [[11](#_bookmark10)]. |
| 23. | self-monitoring | The person keeps a record of one’s own behaviour [[6](#_bookmark5)]. |
| 24. | Delay awards | A process of deferring initial reward for a better reward later [[12](#_bookmark11)]. |
| 25. | Caregiver coping (e.g.,  management skills for the parents of children) | Mechanism through which the caregiver manages the stress [[13](#_bookmark12)]. |
| 26. | Parent-child Interaction Coaching (e.g., positive 1-on-1, attending to children modeling, etc) | The parent applies a new skill with the child and the therapist provides immediate feedback [[14](#_bookmark13)]. |
| 27. | Birth procedures | Knowledge about different ways of giving birth [[15](#_bookmark14)]. |
| 28. | Specific health areas of children (e.g., nutrition, breastfeeding, SRH, etc) | If the intervention targeted the specific dimensions of health areas in children. |
| 29. | Nutrition | Education regarding recommended nutrition practices. |
| 30. | Breastfeeding | Education regarding recommended breastfeeding practices. |
| 31. | Sexual behaviour | Avoidance of risky sexual behaviours. |
| 32. | Identifying thoughts, behaviours, and their links  (e.g., identifying negative thoughts, thought diary, etc.) | Realizing what thoughts cross one’s mind by number of ways [[16](#_bookmark15)]. |
| 33. | Cognitive restructuring (e.g., reattribution, weighing evidence, logical questioning, etc.) | Identifying and disputing maladaptive thoughts [[2](#_bookmark1)]. |
| 34. | Distraction | Paying attention to some other stimuli rather than the unhelpful thoughts [[17](#_bookmark16)]. |
| 35. | Self-talk | Use of self-instruction and self-encouragement to support action [[6](#_bookmark5)]. |
| 36. | Self-praise | To boast one’s self esteem by expressing approval or admiration [[18](#_bookmark17)]. |
| 37. | Mood monitoring | Paying attention toward one's mood states by means of different methods [[19](#_bookmark18)]. |
| 38. | Mindfulness | Paying attention towards experiences in the present moment within body and mind and accepting the happenings [[20](#_bookmark19)]. |
| 39. | Self-awareness | Conscious awareness of becoming the object of one’s own awareness [[21](#_bookmark20)]. |
| 40. | Aerobics | Aerobic exercise also known as "cardio" exercises include running, swimming, walking, hiking, aerobics classes, dancing, cross country skiing, and kickboxing. |
| 41. | Non-aerobic exercise | Anaerobic ("without oxygen") exercise is any physical activity that causes you to be quickly out of breath, like sprinting or lifting a heavy weight. |
| 42. | Motivational enhancement | To increase internal motivation in order to make long lasting change [[22](#_bookmark21)]. |
| 43. | Praise | Praising for positive behaviour or accomplishment. |
| 44. | Role play | Performing role of a person in a situation [[23](#_bookmark22)]. |
| 45. | Behavioural contracting | Agreement of a contract specifying behaviour to be performed so that there is a written record of the person’s resolution witnessed by another [[6](#_bookmark5)]. |
| 46. | Assigning homework | Assigning tasks pertaining to interventions, to be performed at home. |
| 47. | Interpersonal focus | Focus on maintaining relationships with other people [[24](#_bookmark23)]. |
| 48. | Behavioural experiments | To test out the negative thoughts and re-evaluate underlying beliefs by performing an action [[25](#_bookmark24)]. |
| 49. | Motivational interviewing | Prompting the person to provide self-motivating statements and evaluation of their own behaviour to minimize resistance [[6](#_bookmark5)]. |
| 50. | Direct suggestions | Providing directions of how to act, behave or handle a situation [[26,](#_bookmark25) [27](#_bookmark26)]. |
| 51. | Goal setting | Identifying an aim or goal to achieve in a session or therapy [[28](#_bookmark27)]. |
| 52. | Giving sick role | Assigning role of a sick person to the participant, to understand the circumstances, particular rights and responsibilities of those who are ill. |
| 53. | Empathy | To understand and share other’s feeling or situation [[29](#_bookmark28)]. |
| 54. | Macronutrients | Food groups needed in large amounts [[30](#_bookmark29)]. |
| 55. | Micronutrient | Food groups needed in small amounts [[30](#_bookmark29)]. |
| 56. | Eating behaviours | The food choices and eating practices [[31](#_bookmark30)], |
| 57. | Alcohol use | Education about minimal use and harms of alcohol misuse. |
| 58. | Substance misuse | Harmful use of substances for non-medical purposes [[32](#_bookmark31)]. |

**References**

#### Rogers, C.R. and R.E. Farson, *Active listening*. 1957: Industrial Relations Center of the University of Chicago.

1. Chambless, D.L. and S.D. Hollon, *Defining empirically supported therapies.* Journal of consulting and clinical psychology, 1998. **66**(1): p. 7.
2. Marshall, M., et al. *Case management for people with severe mental disorders.* The Cochrane database of systematic reviews, 2000(2): p. CD000050-CD000050.
3. Dovis, S., et al. *Can motivation normalize working memory and task persistence in children with attention-deficit/hyperactivity disorder? The effects of money and computer-gaming.* Journal of abnormal child psychology, 2012. **40**(5): p. 669-681.
4. Dube, S., *Enchantments of modernity: empire, nation, globalization*. 2012: Routledge.
5. Abraham, C. and S. Michie, *A taxonomy of behaviour change techniques used in interventions.* Health psychology, 2008. **27**(3): p. 379.
6. Baran, S.J., E. McDonald, and J. Engberg, *Introduction to mass communication: Media literacy and culture*. 2004: McGraw-Hill.
7. Cregor, M., *The building blocks of positive behaviour.* teaching tolerance, 2008. **34**(4): p. 18-21.
8. Forsyth, J.P., V. Barrios, and D.T. Acheson, *Exposure therapy and cognitive interventions for the anxiety disorders: overview and newer third- generation perspectives*, in *Handbook of exposure therapies*. 2007, Elsevier. p. 61-108.
9. Rolston, A. and E. Lloyd-Richardson, *What is emotion regulation and how do we do it?* Cornell Research Program on Self-Injury and Recovery.[on- line].[cit. 2017-22-09] Available at: [http://www.](http://www/) selfinjury. bctr. cornell. edu/perch/resources/what-is-emotion-regulationsinfo-brief. pdf. Google Scholar, 2017.
10. Janis, I.L. and L. Mann, *Decision making: A psychological analysis of conflict, choice, and commitment*. 1977: free press.
11. Metcalfe, J. and W. Mischel, *A hot/cool-system analysis of delay of gratification: dynamics of willpower.* Psychological review, 1999. **106**(1): p. 3.
12. Zeiss, A.M., et al. *Self-efficacy as a mediator of caregiver coping: Development and testing of an assessment model.* Journal of clinical Geropsychology, 1999. **5**(3): p. 221-230.
13. McNeil, C.B. and T.L. Hembree-Kigin, *Parent-child interaction therapy*. 2010: Springer Science & Business Media.
14. Thompson, R. and Y.D. Miller, *Birth control: to what extent do women report being informed and involved in decisions about pregnancy and birth procedures?* BMC pregnancy and childbirth, 2014. **14**(1): p. 62.
15. Longmore, R.J. and M. Worrell, *Do we need to challenge thoughts in cognitive behaviour therapy?* Clinical psychology review, 2007. **27**(2): p. 173- 187.
16. Fennell, M.J. and J.D. Teasdale, *Effects of distraction on thinking and affect in depressed patients.* British Journal of Clinical Psychology, 1984.

#### **23**(1): p. 65-66.

1. Abdel-Khalek, A.M., *INTRODUCTION TO THE PSYCHOLOGY OF SELF-ESTEEM.* SELF-ESTEEM: p. 1.
2. Swinkels, A. and T.A. Giuliano, *The measurement and conceptualization of mood awareness: Monitoring and labeling one's mood states.* Personality and social psychology bulletin, 1995. **21**(9): p. 934-949.

#### Langer, E.J., *Mindfulness*. 1989: Addison-Wesley/Addison Wesley Longman.

1. Morin, A., *Self‐awareness part 1: Definition, measures, effects, functions, and antecedents.* Social and personality psychology compass, 2011. **5**(10):

#### p. 807-823.

1. Miller, W.R., *Motivational enhancement therapy manual: A clinical research guide for therapists treating individuals with alcohol abuse and dependence*. 1995: DIANE Publishing.
2. Van Ments, M., *The effective use of role-play: Practical techniques for improving learning*. 1999: Kogan Page Publishers.
3. Leary, M.R. and R.F. Baumeister, *The need to belong: Desire for interpersonal attachments as a fundamental human motivation*, in *Interpersonal Development*. 2017, Routledge. p. 57-89.
4. Rouf, K., et al. *Devising effective behavioural experiments.* Oxford guide to behavioural experiments in cognitive therapy, 2004: p. 21-58.
5. Rossi, E.L. and K.L. Rossi, *What is a suggestion? The neuroscience of implicit processing heuristics in therapeutic hypnosis and psychotherapy.*

#### American Journal of Clinical Hypnosis, 2007. **49**(4): p. 267-281.

1. Podikunju-Hussain, S., *Working with Muslims: Perspectives and suggestions for counseling.* Vistas: Compelling perspectives on counseling, 2006: p. 103-106.
2. Locke, E.A. and G.P. Latham, *A theory of goal setting & task performance*. 1990: Prentice-Hall, Inc.
3. Chinsky, J.M. and J. Rappaport, *Brief critique of the meaning and reliability of" accurate empathy" ratings.* Psychological Bulletin, 1970. **73**(5): p. 379.
4. Hadigan, C.M., et al. *Assessment of macronutrient and micronutrient intake in women with anorexia nervosa.* International Journal of Eating Disorders, 2000. **28**(3): p. 284-292.
5. Turconi, G., et al. *Eating habits and behaviours, physical activity, nutritional and food safety knowledge and beliefs in an adolescent Italian population.* Journal of the American College of Nutrition, 2008. **27**(1): p. 31-43.
6. Lingford-Hughes, A., S. Welch, and D. Nutt, *Evidence-based guidelines for the pharmacological management of substance misuse, addiction and comorbidity: recommendations from the British Association for Psychopharmacology.* Journal of Psychopharmacology, 2004. **18**(3): p. 293-335

**Supplementary Table 5: Characteristics of studies delivered to individuals (n=24)**

| **Reference** | **Outcome assessment** | **Timing of delivery** | **Setting** | **Intervention focus** | **Nature** | **Delivery agent** | **Integration into the healthcare setting** | **Number of sessions** | **Duration of one session (min)** | **Duration of overall program (weeks)** | **Booster sessions** |
| --- | --- | --- | --- | --- | --- | --- | --- | --- | --- | --- | --- |
| Burns, 2013 | EPDS | Antenatal | Home visits | Treatment | CBT | Two therapists, one with master’s level experience and the other with doctoral experience in CBT | No | 12 | NR | 12-15 weeks | NR |
| Chabrol, 2002, Prev | EPDS | Postpartum | Home visits | Prevention | CBT | psychology graduate | No | 1 | 60 | NR | NR |
| Chabrol, 2002, Treat | HDRS | Postpartum | Home visits | Treatment | CBT | psychology graduate | No | 5to 8 | NR | 5 to 8 | NR |
| Cho, 2008 | BDI | Antenatal | Clinic | Prevention | CBT | clinical psychologist | No | 9 | 60 | 18 | NR |
| Cooper, 2003 | EPDS | Postpartum | Home visits | Treatment | CBT | three non-specialists and one specialist | No | 10 | NR | 10 | NR |
| Dimidjian, 2016 | EPDS | Antenatal | Hospital | Prevention | MBCT-PD | Psychologists, behavioural health scientists | Yes | 8 | 120 | 8 | Optional once a month |
| Dimidjian, 2017 | PHQ-9 | Antenatal | Mixed | Treatment | BA | Among non-specialists, four had a nursing degree (nurse midwife, nurse practitioner), three had a master’s degree in behavioural health, and one was a registered occupational therapist. All were naïve to BA at the outset of the study. | Yes | 10 | NR | NR | NR |
| Hou, 2014 | EPDS | Postpartum | Clinic | Treatment | CBT | Psychological counsellors | Yes | 13 (plus 6 SFT sessions to family) | 60 (60-90 minutes for SFT sessions) | 13 | NR |
| Morrell, 2009 | EPDS | Postpartum | Home visits | Prevention | CBA | Health visitors | Yes | 8 | 60 | 8 | NR |
| Nasiri, 2018 | BDI | Postpartum | Healthcare centre | Treatment | PST | Researcher | No | 6 | 45-50 | 6 | NR |
| Nejad, 2021 | DASS-21 | Antenatal | Healthcare centre | Treatment | MBSR | Mental health midwife | No | 8 | 120 | 8 | NR |
| Ngai, 2015 | EPDS >= 10 | Postpartum | Hospital | Treatment | CBT | Midwives | Yes | NR | NR | 5 | NR |
| O'Mahen, 2013 | BDI-II | Both | Hospital | Treatment | CBT | Masters and doctoral level social workers and psychologists | No | 12 | 55 | 12 | NR |
| Tandon, 2018 | BDI-II | Both | Home visits | Prevention | CBT | Home visitors | Yes | 15 | 20 |  | NR |
| Trevillion, 2016 | EPDS | Both | Clinic | Treatment | CBT | Psychological wellbeing practitioner | Yes | 6 | 45 | 6 | NR |
| Van Horne, 2021 | EPDS | Postnatal | Home visits | Treatment | PST | Social worker | Yes | 5 | 60 | 5 | NR |
| Yazdanimehr, 2016 | EPDS | Antenatal | Healthcare center | Treatment | MCBT | Clinical psychologist | No | 8 | 90 | 8 |  |
| Rahman, 2008 | HDRS | Both | Home visits | Treatment | CBT | Lady Health Workers | Yes | 16 | NR | 11 | NR |
| Silverstein, 2011 | QIDS | Antenatal | Mixed | Prevention | PST | Educators: Four graduate students—pursuing degrees in social work, public health, and graduate medical sciences | No | 4 | NR | NR | NR |
| Ammerman, 2013 | EPDS | Antenatal | Home visits | Treatment | CBT | Social worker | Yes | 15 | 60 | 15 | 1 |
| McKee, 2006 | BDI-II | Both | Mixed | Treatment | CBT | Social worker | No | 8 | NR | NR | NR |
| Prendergast, 2001 | EPDS | Both | Home visits | Treatment | CBT | Early childhood nurse | Yes | 6 | 60 | 6 | NR |
| Hayden, 2012 | BDI | Antenatal | Clinic | Treatment | CBT | Clinical social workers | No | NR | NR | NR | NR |
| Sikander, 2019 | PHQ-9 | Both | Home visits | Treatment | CBT | Volunteer peers | Yes | 14 | 45 | 14 | NR |

**Supplementary Table 6: Characteristics of interventions delivered to group (n=25)**

| **Reference** | **Outcome assessment** | **Timing of delivery** | **Setting** | **Intervention focus** | **Nature** | **Delivery agent** | **Integration into the healthcare setting** | **Number of sessions** | **Duration of one session (min)** | **Duration of overall program (weeks)** | **Booster sessions** |
| --- | --- | --- | --- | --- | --- | --- | --- | --- | --- | --- | --- |
| Jesse, 2015 low risk | EPDS | Antenatal | Hospital | Treatment | CBT | Licensed clinical social workers and other MHPs | Yes | 6 | 120 | 6 | NR |
| Jesse, 2015 high risk | EPDS | Antenatal | Hospital | Treatment | CBT | Licensed clinical social workers and other MHPs | Yes | 6 | 120 | 6 | NR |
| Kaaya, 2013 | Hopkins symptoms checklist | Antenatal | Clinic | Prevention | PST | Social worker/Psychiatric nurses | No | 6 | NR | 6 | NR |
| Khamseh, 2019 | BDI | Antenatal | Clinic | Treatment | PST | Researcher | Yes | 5 | 90 | 5 | NR |
| Le, 2011 | BDI-II | Antenatal | Clinic | Prevention | CBT | Research Assistants ( post bachelors trained) | No | 8 | 120 |  | 3 |
| Leung, 2013 | EPDS | Antenatal | Clinic | Prevention | CBT | non-specialists | No | 6 | 120 | 6 | NR |
| Milgrom, 2005 | BDI | Postpartum | Clinic | Treatment | CBT | Therapist and co-therapist (co-therapists with professional registrations and backgrounds in clinical psychology, postgraduate psychology research, and nursing with postgraduate qualifications in counselling and/or psychology) | No | 9 | 90 | 12 | NR |
| Ngai, 2020 couple therapy | EPDS | Both | Clinic | Treatment | CBT | experienced midwife | No | 1 | 180 |  | Yes |
| Ngai, 2020 women alone |  | Both | Clinic | Treatment | CBT | experienced midwife | No |  |  |  | NR |
| Tandon, 2014 | BDI-II | Both | Home visits | Prevention | CBT | Social workers or clinical psychologists | No | 6 | 120 | 6 | 2 |
| Van Lieshout, 2022 | EPDS | Postnatal | Community | Treatment | CBT | Registered nurse | No | 9 | 120 | 9 | NR |
| Van Ravesteyn, 2018 | EPDS | Antenatal | Clinic | Treatment | CBT | Social psychiatric nurse; perinatal psychiatrist, clinical psychologist, Infant Mental Health Specialist, creative arts therapist, | Yes | 5 | 360/6 hrs | 5 | NR |
| Zemestani, 2020 | BDI-II | Antenatal | Healthcare centre | Treatment | MCBT | Clinical psychologist | No | 8 | 120 | 8 | NR |
| Futterman, 2010 | CES-D | Antenatal | Clinic | Prevention | CBT | Peers/Mothers with HIV, having a child, attended M2M programme previously | Yes | 8 | NR | 8 | NR |
| Kozinszky, 2012 | Leverton questionnaire | Antenatal | Clinic | Prevention | CBT | Health visitors | Yes | 4 | NR | 4 | NR |
| Lara, 2010 | SCID | Antenatal | Hospital | Prevention | Psychoeducation | Clinicians | Yes | 8 | 120 | 8 | NR |
| Mao, 2012 | PHQ-9 | Postnatal | Hospital | Prevention | CBT | Obstetrician | Yes | 4 | 90 | 4 | NR |
| Muñoz, 2007 | CES-D | Both | Home visits | Prevention | CBT | Led by facilitators were faculty, postdoctoral fellows, and advanced doctoral graduate students in clinical psychology | No | 12 | NR | 12 | 4 |
| Leung, 2016 | EPDS | Postnatal | Clinic | Treatment | CBT | Non-specialists | Yes |  | 120 | 6 | NR |
| Puckering, 2010 | EPDS | Postnatal | Clinic | Treatment | CBT | Counselling psychologist | Yes | 14 | 360 | 14 | NR |
| Rojas, 2007 | EPDS | Postnatal | Clinic | Treatment | Psychoeducation | Midwives or nurses, or doctors | Yes | 8 | 50 | 8 | NR |
| Brugha, 2000a | EPDS | Antenatal | Clinic | Prevention | CBT | Nurses, Occupational therapists | Yes | 6 | 120 | 6 | 1 |
| Austin, 2008 | EPDS | Antenatal | Clinic | Prevention | CBT | Clinical psychologist, assisted by a midwife | No | 6 | 120 | 6 | 1 |
| Bittner, 2014 | EPDS | Antenatal | Clinic | Prevention | CBT | Clinical psychologists | No | 8 | 90 | NR | NR |
| Hagan, 2004 | EPDS | Postpartum | Clinic | Treatment | CBT | Midwives | No | 6 | 120 | 6 | NR |

**Supplementary Table 7: Characteristics of interventions delivered online (n=10)**

| **Reference** | **Outcome assessment** | **Timing of delivery** | **Intervention focus** | **Nature** | **Delivery agent** | **Format** | **Integration into the healthcare setting** | **Number of sessions** | **Duration of one session (min)** | **Duration of overall program (weeks)** | **Booster sessions** |
| --- | --- | --- | --- | --- | --- | --- | --- | --- | --- | --- | --- |
| Duffecy, 2019 | PHQ-9 | Antenatal | Prevention | CBT | Online | Group | No | 16 | 10 to 15 | 8 | 3 |
| Fonseca, 2020 | EPDS | Postpartum | Prevention | CBT-ACT | Online | Individual | No | 5 | NR | 5 | NR |
| Forsell, 2017 | EPDS | Antenatal | Treatment | CBT | Online | Individual | Yes | 10 | NR | NR | NR |
| Jannati, 2020 | EPDS | Postpartum | Treatment | CBT | self-help | Individual | No | 8 | 45-60 | 8 | NR |
| Loughnan, 2019 | PHQ-9 | Postpartum | Treatment | CBT | self-help | Individual | No | 3 | NR | 6 | NR |
| Loughnan, 2019 | PHQ-9 | Antenatal | Treatment | CBT | Online | Individual | No | 3 | NR | 6 | NR |
| Milgrom, 2016 | BDI-II | Postpartum | Treatment | CBT | website | Individual | No | 6 | NR | 6 | NR |
| O'Mahen, 2013 | EPDS | Postpartum | Treatment | BA | website | Individual | No | 11 | 40 | 14 | NR |
| Van Lieshout, 2021 | EPDS | Postpartum | Treatment | CBT | Facilitated by psychotherapist, psychiatrist, psychology graduate student | Group | No | 1 | 420 | 1 | NR |
| Wozney, 2017 | BDI-II | Postpartum | Treatment | CBT | Facilitated by paraprofessionals | Individual | No | 12 | NR | 12 | NR |

**Supplementary Table 8: Subgroup analyses for categorical intervention and participant level characteristics**

| **Group** | **n** | **SMD** | **95% CI** | | **Q** | **df (Q)** | **p** | **I** |
| --- | --- | --- | --- | --- | --- | --- | --- | --- |
| **Scope of intervention** | | | | | | | | |
| Prevention | 20 | -0.36 | -0.65 | -0.07 | 9.96 | 1.00 | <0.001 | 47.23 |
| Treatment | 38 | -0.94 | -1.15 | -0.73 |  |  |  | 94.48 |
| **Timing of intervention** | | | | | | | | |
| Antenatal | 26 | -0.63 | -0.91 | -0.36 | 1.95 | 2 | 0.38 | 84.07 |
| Both | 11 | -0.66 | -1.06 | -0.25 |  |  |  | 96.58 |
| Postnatal | 21 | -0.90 | -1.19 | -0.61 |  |  |  | 92.78 |
| **Mode of delivery** | | | | | | | | |
| Electronic | 9 | -1.19 | -1.64 | -0.75 | 4.76 | 2 | 0.09 | 93.99 |
| Group | 25 | -0.66 | -0.92 | -0.40 |  |  |  | 94.59 |
| Individual | 24 | -0.65 | -0.92 | -0.39 |  |  |  | 80.99 |
| **Theoretical orientation** | | | | | | | | |
| BA | 2 | -0.33 | -1.22 | 0.57 | 24.17 | 8.00 | <0.001 | 79.26 |
| CBA | 1 | -0.35 | -1.62 | 0.91 |  |  |  | 0.00 |
| CBT | 43 | -0.69 | -0.90 | -0.49 |  |  |  | 93.59 |
| CBT-ACT | 1 | -0.40 | -1.69 | 0.90 |  |  |  | 0.00 |
| MBCT-PD | 1 | -0.24 | -1.59 | 1.11 |  |  |  | 0.00 |
| MBSR | 1 | -1.68 | -3.05 | -0.32 |  |  |  | 0.00 |
| MCBT | 2 | -3.26 | -4.37 | -2.16 |  |  |  | 94.12 |
| PST | 5 | -0.71 | -1.31 | -0.11 |  |  |  | 82.31 |
| Psychoeducation | 2 | -0.59 | -1.52 | 0.33 |  |  |  | 0.00 |
| **Delivery agent** | | | | | | | | |
| Multidisciplinary | 6 | -0.28 | -0.79 | 0.24 | 7.18 | 3 | 0.07 | 0.00 |
| Non-specialists | 22 | -0.61 | -0.88 | -0.34 |  |  |  | 94.91 |
| Online | 8 | -1.09 | -1.57 | -0.62 |  |  |  | 90.81 |
| Specialist | 22 | -0.89 | -1.18 | -0.61 |  |  |  | 89.80 |
| **Integrated into health system** | | | | | | | | |
| No | 33 | -1.01 | -1.24 | -0.79 | 14.07 | 1 | <0.001 | 94.76 |
| Yes | 25 | -0.38 | -0.63 | -0.14 |  |  |  | 56.90 |
| **Format of delivery** | | | | | | | | |
| Group | 24 | -0.74 | -1.02 | -0.46 | 0.18 | 2 | 0.91 | 95.94 |
| Individual | 31 | -0.76 | -1.00 | -0.51 |  |  |  | 85.49 |
| Mixed | 3 | -0.58 | -1.35 | 0.19 |  |  |  | 0.00 |

**Supplementary Table 9: Bivariate meta-regression analyses for continuous intervention and participant level characteristics**

| **Covariate** | **Coefficient** | **SE** | **95% CI** | | **Z-value** | **P-value** |
| --- | --- | --- | --- | --- | --- | --- |
| **Maternal age** | | | | | | |
| **Intercept** | 1.54 | 0.24 | 1.06 | 2.02 | 6.34 | <0.01 |
| **maternal age** | -0.07 | 0.01 | -0.09 | -0.06 | -8.46 | <0.01 |
| **Proportion of married women** | | | | | | |
| **Intercept** | -0.3 | 0.07 | -0.43 | -0.17 | -4.52 | <0.01 |
| **Married** | -0 | 0 | -0 | 0 | -1.91 | 0.06 |
| **Proportion of participants from minority ethnic groups** | | | | | | |
| **Intercept** | -0.705 | 0.065 | -0.832 | -0.577 | -10.81 | <0.01 |
| **Ethnicity** | 0.004 | 0.001 | 0.001 | 0.007 | 2.66 | 0.01 |
| **Proportion of primiparous women** | | | | | | |
| **Intercept** | -0.441 | 0.071 | -0.579 | -0.302 | -6.24 | <0.01 |
| **Primiparous women** | 0 | 0.001 | -0.002 | 0.003 | 0.25 | 0.8 |
| **Proportion of participants with low-income levels** | | | | | | |
| **Intercept** | -1.03 | 0.115 | -1.255 | -0.804 | -8.95 | <0.01 |
| **Low income** | 0.009 | 0.002 | 0.004 | 0.013 | 3.47 | <0.01 |
| **Proportion of participants with poor education** | | | | | | |
| **Intercept** | -0.784 | 0.057 | -0.896 | -0.671 | -13.67 | <0.01 |
| **Poor education** | 0.006 | 0.001 | 0.005 | 0.008 | 6.43 | <0.01 |
| **Proportion of participants with past history of mental health problems** | | | | | | |
| **Intercept** | -0.583 | 0.126 | -0.83 | -0.336 | -4.62 | 0 |
| **History of depression** | 0.004 | 0.002 | 0 | 0.008 | 2.15 | 0.03 |

**Supplementary Figure 1: Non-specific ingredients in included trials testing interventions delivered to individuals (n= 24)**

**
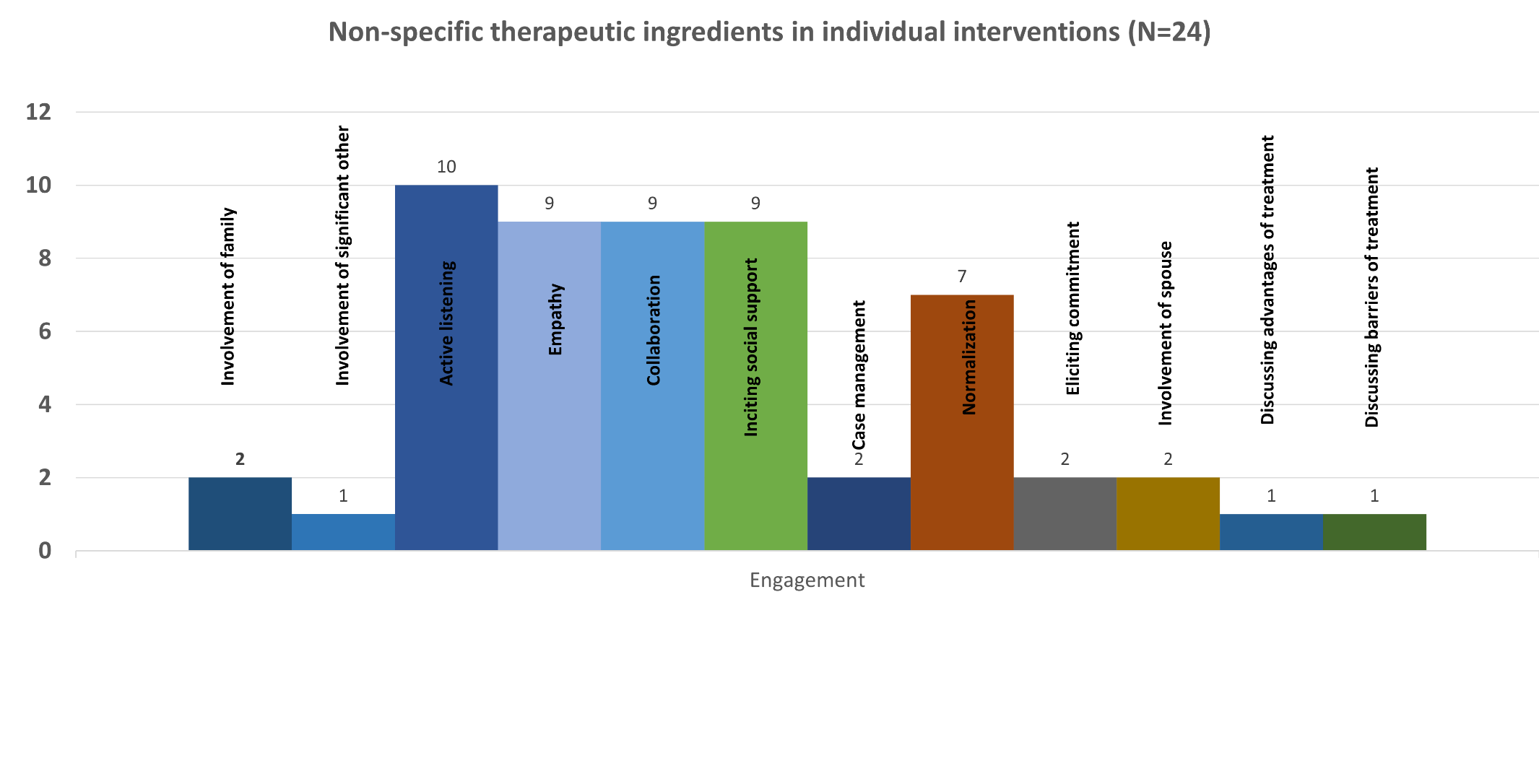
**

**Supplementary Figure 2: In-session delivery techniques utilized in included trials testing interventions delivered to individuals (n= 24)**

**
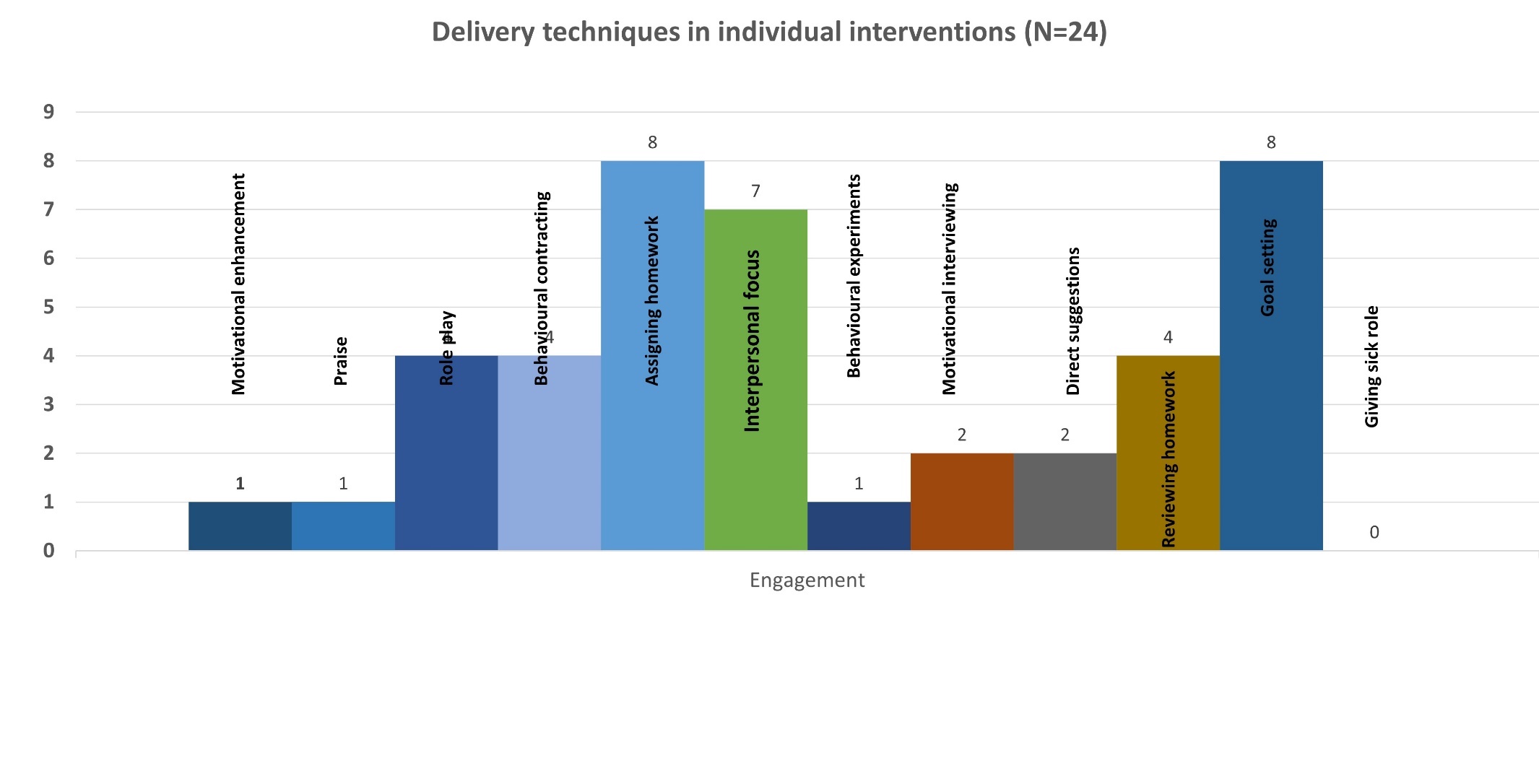
**

**Supplementary Figure 3: Specific ingredients utilized in included trials testing interventions delivered to individuals (n= 24)**

**
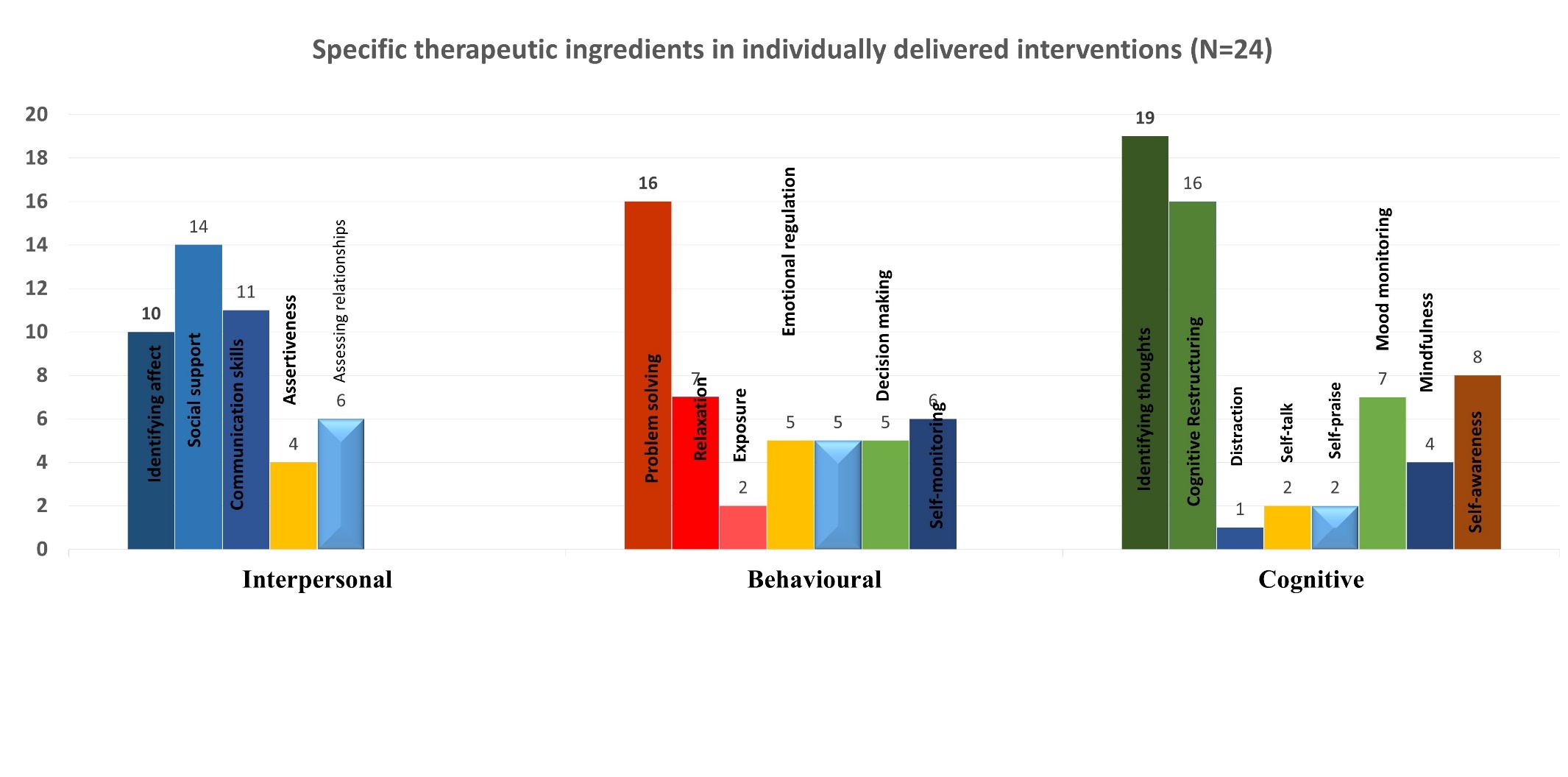
**

**Supplementary Figure 4: Non-specific ingredients utilized in included trials testing interventions delivered to groups (n= 25)**

**
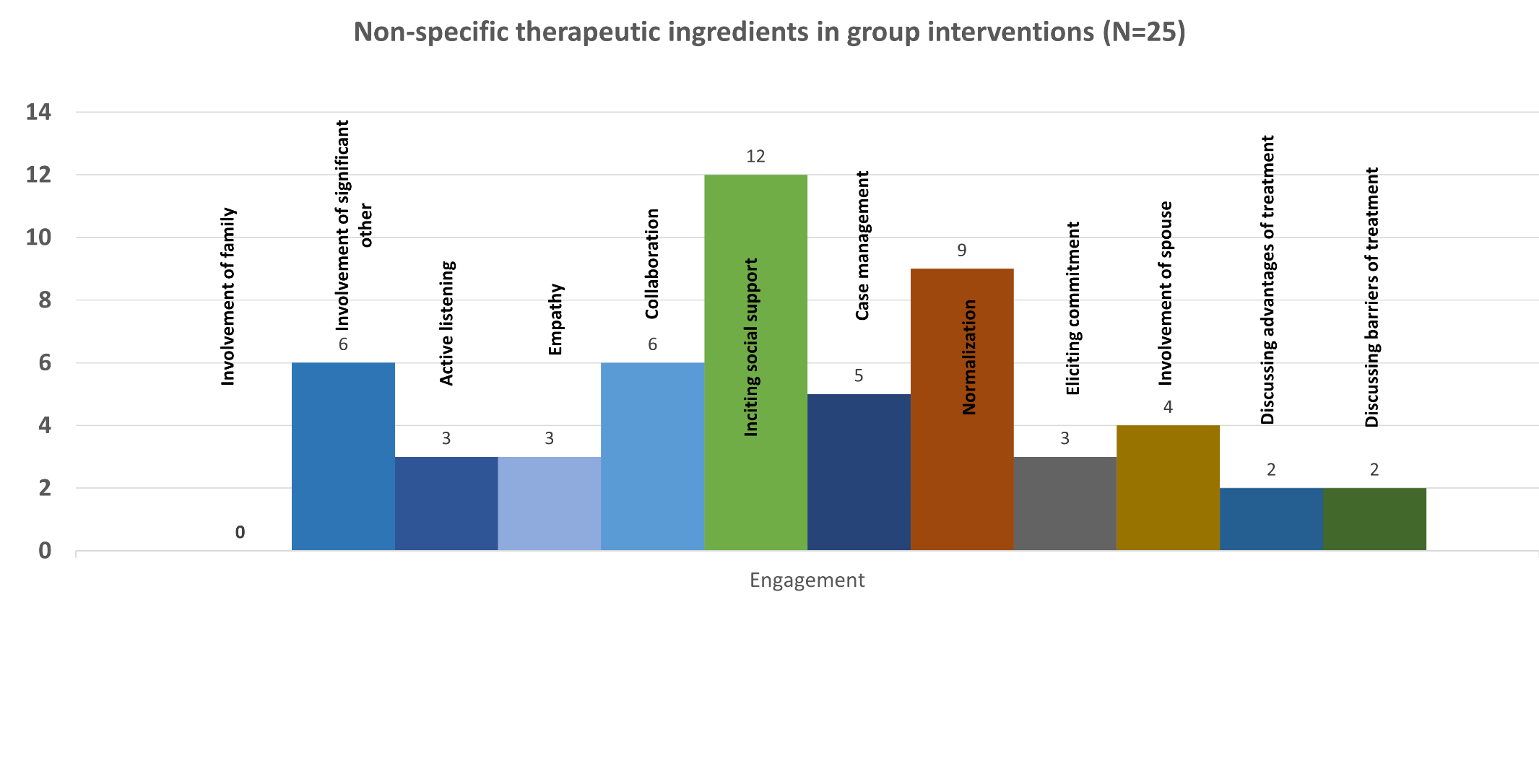
**

**Supplementary Figure 5: In-session techniques utilized in included trials testing interventions delivered to groups (n= 25)**

**
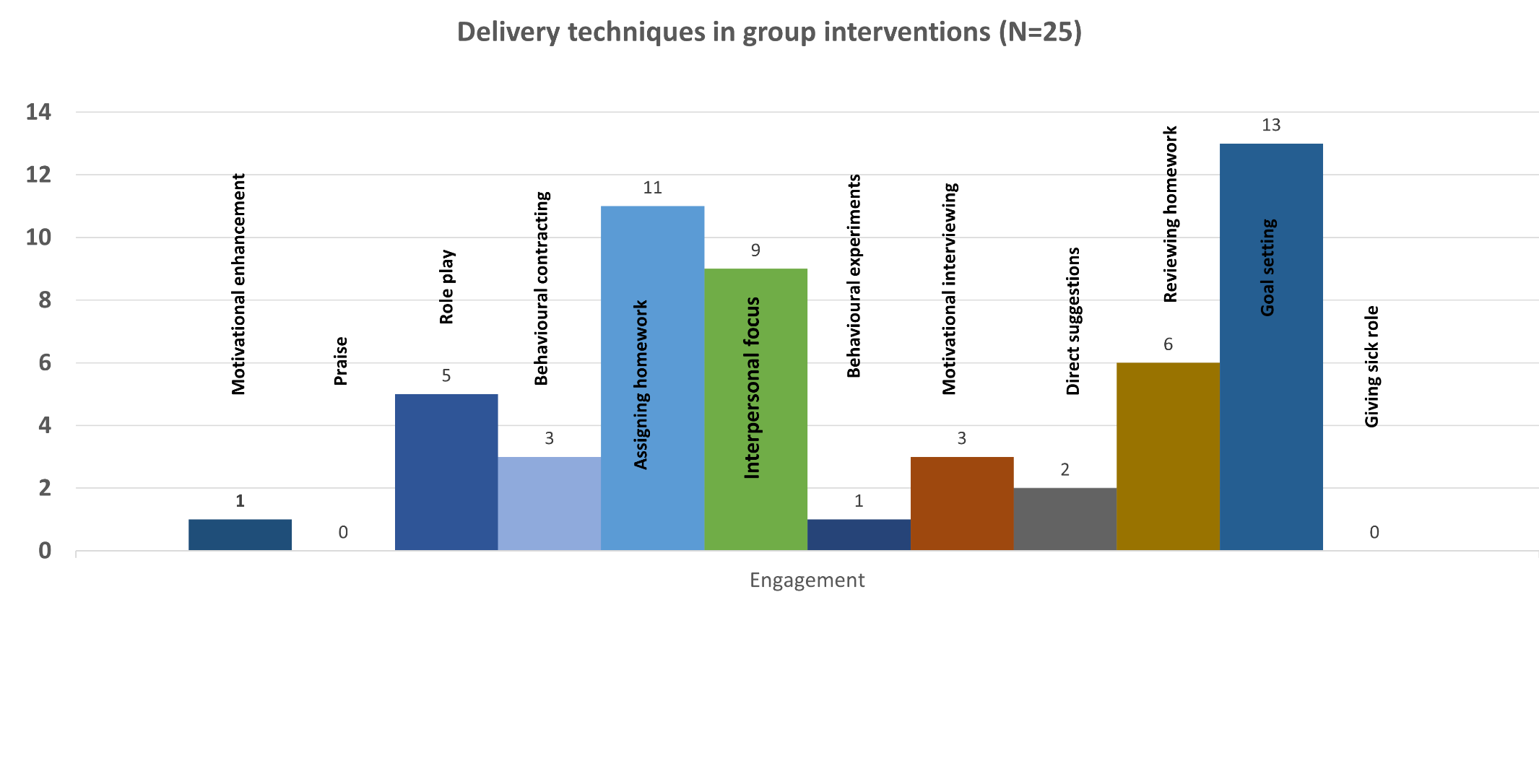
**

**Supplementary Figure 6: Specific ingredients utilized in included trials testing interventions delivered to groups (n= 25)**

**
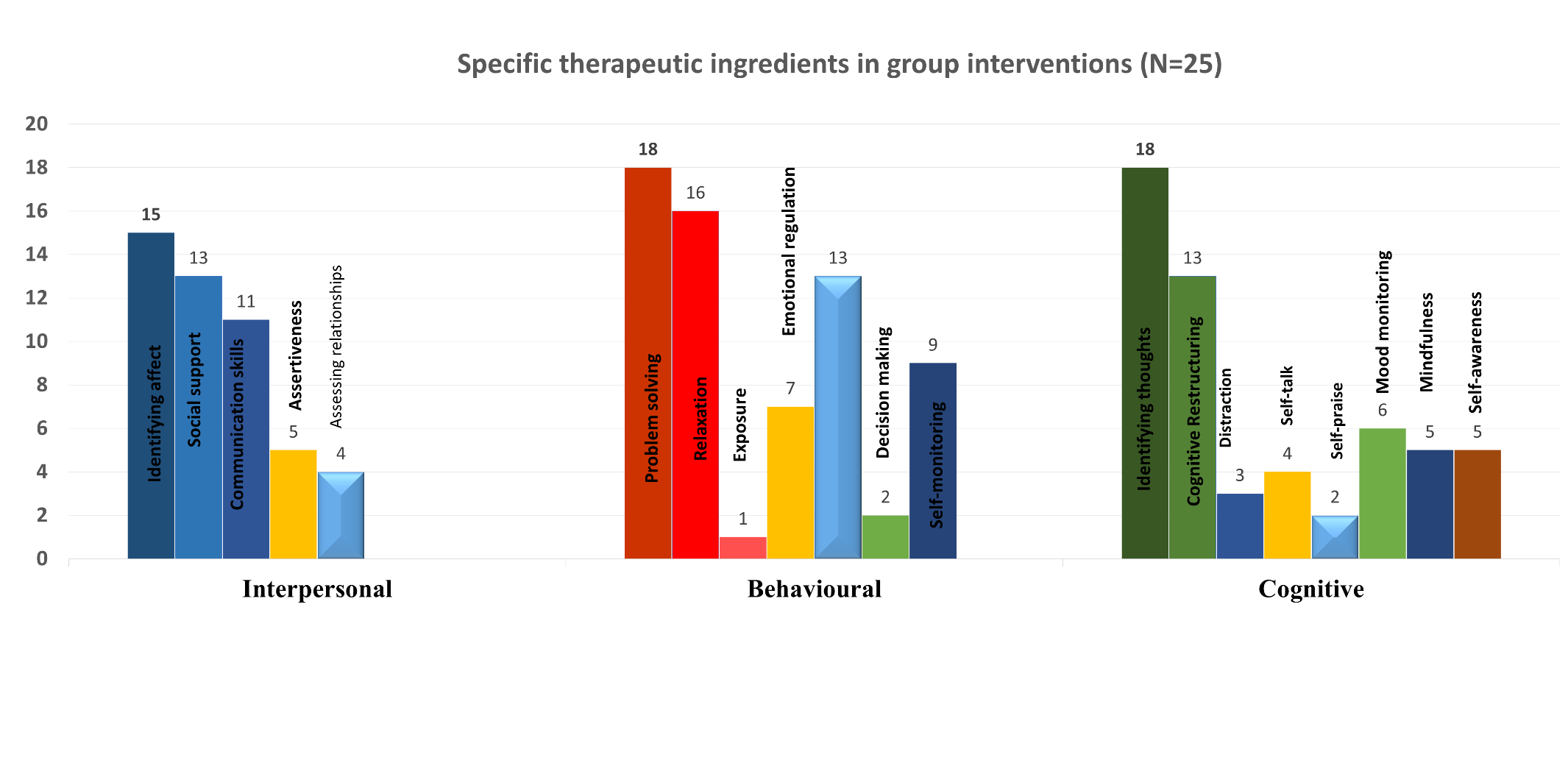
**

**Supplementary Figure 7: Non-specific ingredients utilized in included trials testing interventions delivered electronically (n= 10)**

**
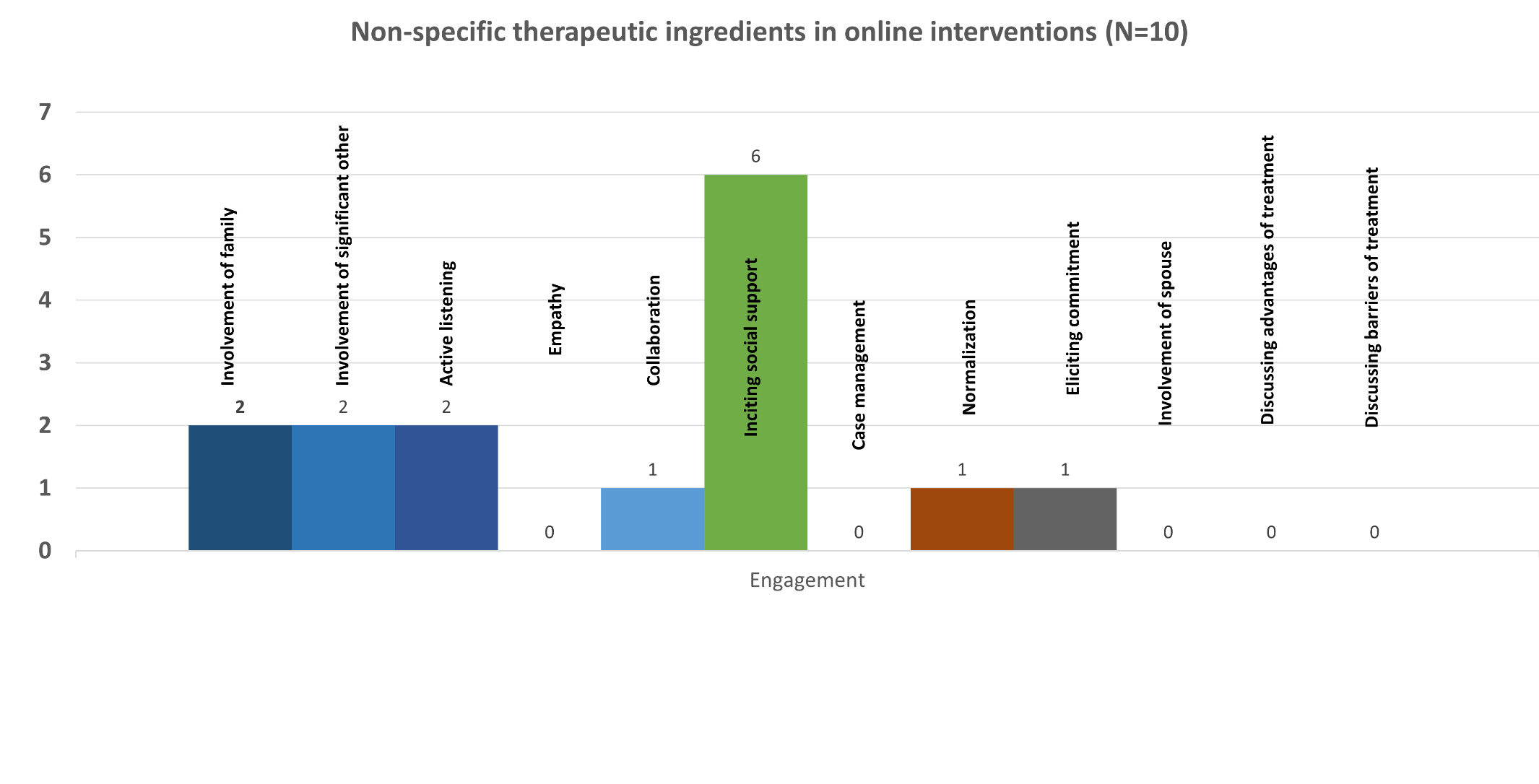
**

**Supplementary Figure 8: Delivery techniques utilized in included trials testing interventions delivered electronically (n= 10)**

**
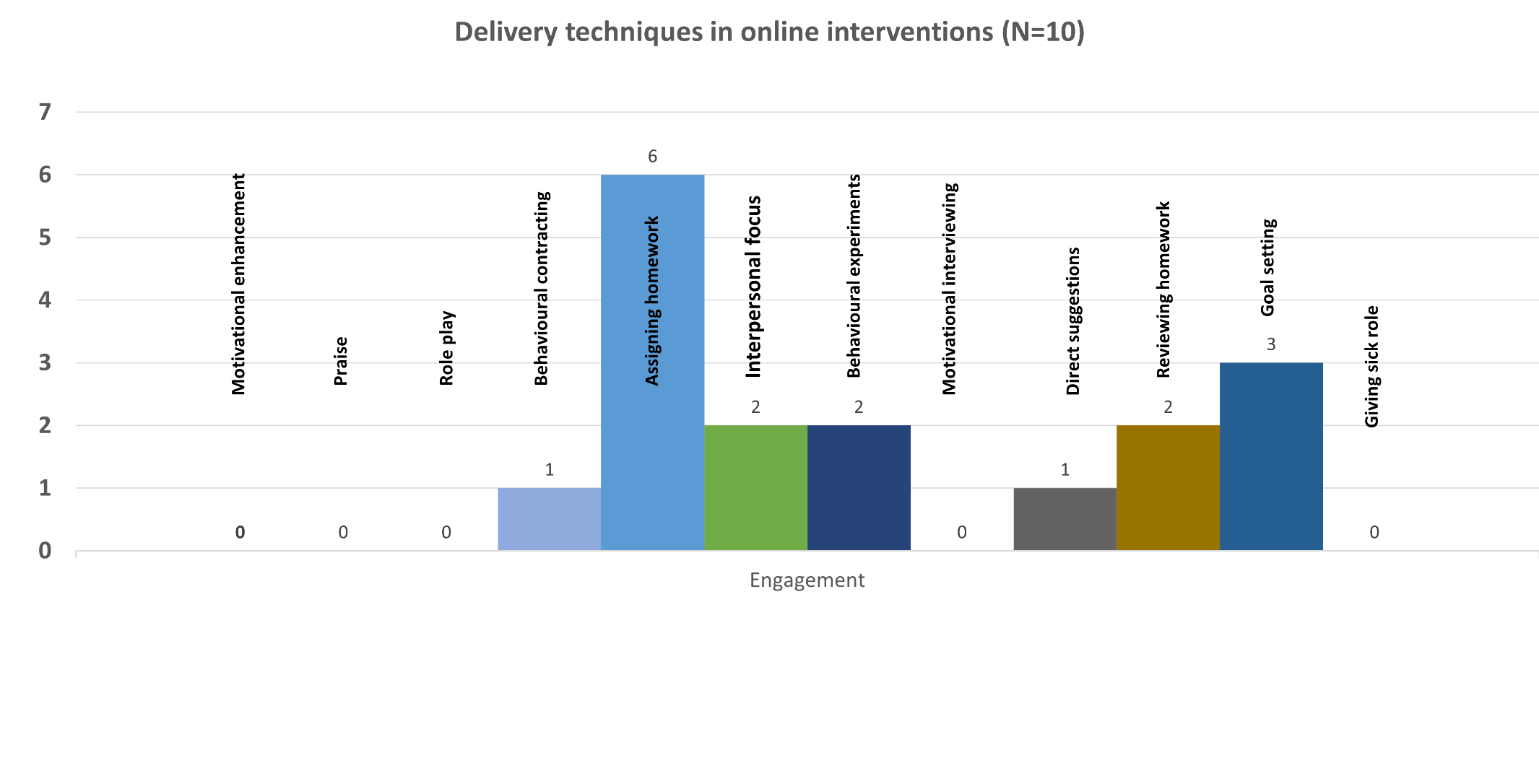
**

**Supplementary Figure 9: Specific ingredients utilized in included trials testing interventions delivered electronically (n= 10)**

**
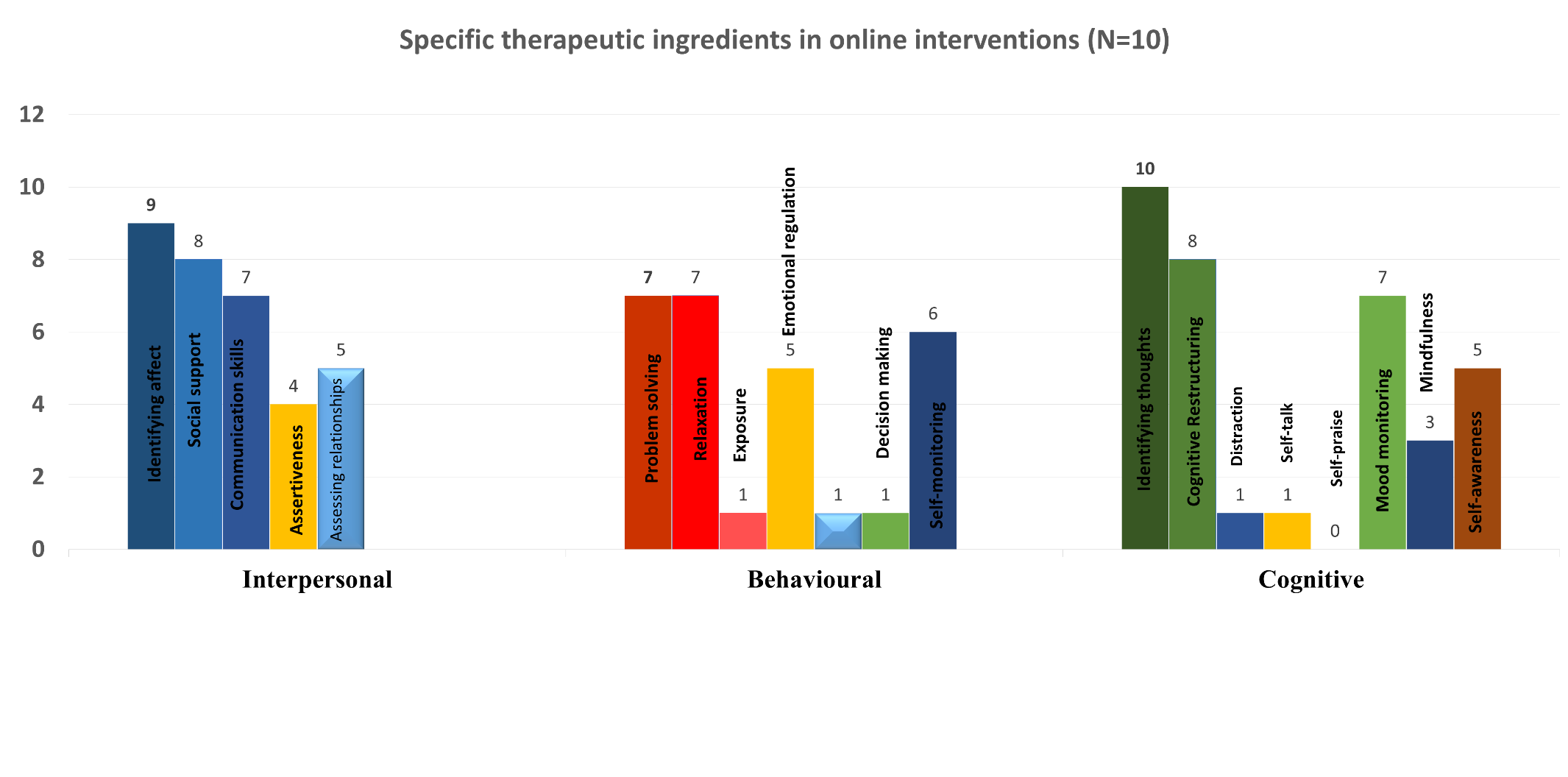
**

**Supplementary Figure 10: Funnel plot visualizing publication bias among studies included in the meta-analysis**
